# Identification of dual-purpose therapeutic targets implicated in aging and glioblastoma multiforme using PandaOmics - an AI-enabled biological target discovery platform

**DOI:** 10.18632/aging.204678

**Published:** 2023-04-26

**Authors:** Andrea Olsen, Zachary Harpaz, Christopher Ren, Anastasia Shneyderman, Alexander Veviorskiy, Maria Dralkina, Simon Konnov, Olga Shcheglova, Frank W. Pun, Geoffrey Ho Duen Leung, Hoi Wing Leung, Ivan V. Ozerov, Alex Aliper, Mikhail Korzinkin, Alex Zhavoronkov

**Affiliations:** 1 The Youth Longevity Association, Sevenoaks, NA, United Kingdom; 2 Pine Crest School Science Research Department, Fort Lauderdale, Florida 33334, USA; 3 Shanghai High School International Division, Shanghai 200231, China; 4 Insilico Medicine Hong Kong Ltd., Hong Kong Science and Technology Park, New Territories, Hong Kong, China

**Keywords:** aging, target discovery, GBM, glioblastoma, PandaOmics

## Abstract

Glioblastoma Multiforme (GBM) is the most aggressive and most common primary malignant brain tumor. The age of GBM patients is considered as one of the disease's negative prognostic factors and the mean age of diagnosis is 62 years. A promising approach to preventing both GBM and aging is to identify new potential therapeutic targets that are associated with both conditions as concurrent drivers. In this work, we present a multi-angled approach of identifying targets, which takes into account not only the disease-related genes but also the ones important in aging. For this purpose, we developed three strategies of target identification using the results of correlation analysis augmented with survival data, differences in expression levels and previously published information of aging-related genes. Several studies have recently validated the robustness and applicability of AI-driven computational methods for target identification in both cancer and aging-related diseases. Therefore, we leveraged the AI predictive power of the PandaOmics TargetID engine in order to rank the resulting target hypotheses and prioritize the most promising therapeutic gene targets. We propose cyclic nucleotide gated channel subunit alpha 3 (*CNGA3*), glutamate dehydrogenase 1 (*GLUD1*) and sirtuin 1 (*SIRT1*) as potential novel dual-purpose therapeutic targets to treat aging and GBM.

## INTRODUCTION

Glioblastoma Multiforme (GBM) is the most deadly and aggressive brain tumor that exists, accounting for 16% of all primary brain tumors [1]. GBM is caused by genetic mutations leading to uncontrolled growth of glial stem or progenitor cells. This cancer is divided into primary GBM (*de novo*) and secondary GBM (progression from lower-grade tumors) [2]. Secondary GBM occurs most often in younger patients with a mean age of 45 years, whereas primary GBM most often happens in patients with a mean age of 62 years. Secondary GBM is, furthermore, the predominant GBM diagnosis in pediatric cases [1]. Primary GBM differs from secondary glioblastomas in its mutations, proteins, and pathways involved, as well as the distribution of molecular subtypes among patients (Proneural, Neural, Classical, Mesenchymal) [3, 4].

Current treatment for GBM includes maximal surgical resection, radiotherapy, and chemotherapy, such as concomitant and maintenance temozolomide, and these approaches usually lead to tumor resurgence and a median survival of 15 months [5]. Recently, tumor-treating fields (TTF), was proposed as a novel glioblastoma therapy that uses alternating electric fields to disrupt the division of cancer cells. TTF therapy has been shown to improve overall survival in patients with newly diagnosed or recurrent glioblastoma when used in combination with standard treatments such as chemotherapy and radiation therapy [6]. It is important to highlight some of the immunotherapies are either already approved for the treatment of GBM or on the way to being approved. For example, Pembrolizumab and Nivolumab are two checkpoint inhibitors that are being tested in clinical trials for glioblastoma [7]. However, despite significant advances in treating glioblastoma, the current standard of care for GBM is damaging to the brain and results in an overall 10-year survival rate (after the diagnosis) of 0.71% [8].

Most targeted therapeutics for GBM account for 1 to 2 targets in the GBM tumor, such as epidermal growth factor receptor (EGFR) and isocitrate dehydrogenase (IDH1), allowing cancer cells without expressing specific targets to survive and eventually kill the patient [9, 10]. More genetic targets for GBM must be identified and a method to personalize gene therapy for GBM patients may be useful for treating the many varieties of mutations present in GBM. Furthermore, identifying potentially effective combinations of drugs for the treatment of GBM patients using automated drug discovery approaches is an important task [11].

Due to the variety of mutations and development of GBM across different ages [3], this study utilized artificial intelligence (AI) and age-correlation analysis with the goal to identify dual-purpose targets associated with aging and GBM leveraging the power of AI-driven PandaOmics platform, which integrates analysis of multi-omics and literature data. Recently, the platform has been demonstrated to predict novel age-associated targets for the purpose of drug discovery [12].

Identifying therapeutic targets is crucial for successful drug development, as erroneous targets can lead to costly programs and failed clinical trials. While traditional computational approaches for target and biomarker discovery are important for success, they face limitations due to complex data and batch effects [13]. However, AI-driven approaches have shown efficacy in this area, using pathway analysis and algorithms on multiomics data to identify new targets and biomarkers, even with insufficient prior evidence [14–17].

Insilico Medicine scientists have pioneered the use of generative artificial intelligence in biology since 2016 [18, 19]. Many of the summary of the original generative biology approaches utilizing the generative systems for generation of synthetic biological data and the first demonstration of PandaOmics platform were first presented at the Interdisciplinary Workshop and the National Institute of Aging [20]. In addition to the target discovery using generative biology approaches, Insilico developed the capabilities in generative chemistry with the first peer reviewed publication in 2016 [21–24]. These approaches have been successfully applied to various diseases and have demonstrated their potential in identifying novel compounds and accelerating drug development [25, 26]. As such, generative chemistry and biology approaches are becoming increasingly important for the current and future drug discovery efforts. This paper demonstrates the application of generative biology approach to the complex interplay between GBM and Aging.

Aging is the exponential decline in homeostatic capabilities, which ultimately leads to an increased risk of age-related diseases and death [27]. With the rise of the longevity field, a novel approach has been undertaken to target diseases not just separately, with the goal to slow aging, but multiple diseases at once. Aging has, furthermore, been studied in the context of specific diseases, as we did in the present study. GBM has particularly strong aging-related effects on patients (including decreased resilience for aggressive treatment, comorbidity, and age-dependent immune system status), at the same time, this disease is prompted by old age [28]. By investigating GBM from both the traditional cancer-targeting approach and with the novel aging-oriented approach, we hope to find new genetic targets, which may alleviate both the onset of GBM as well as slow aging in patients.

## RESULTS

**Figure 1:**
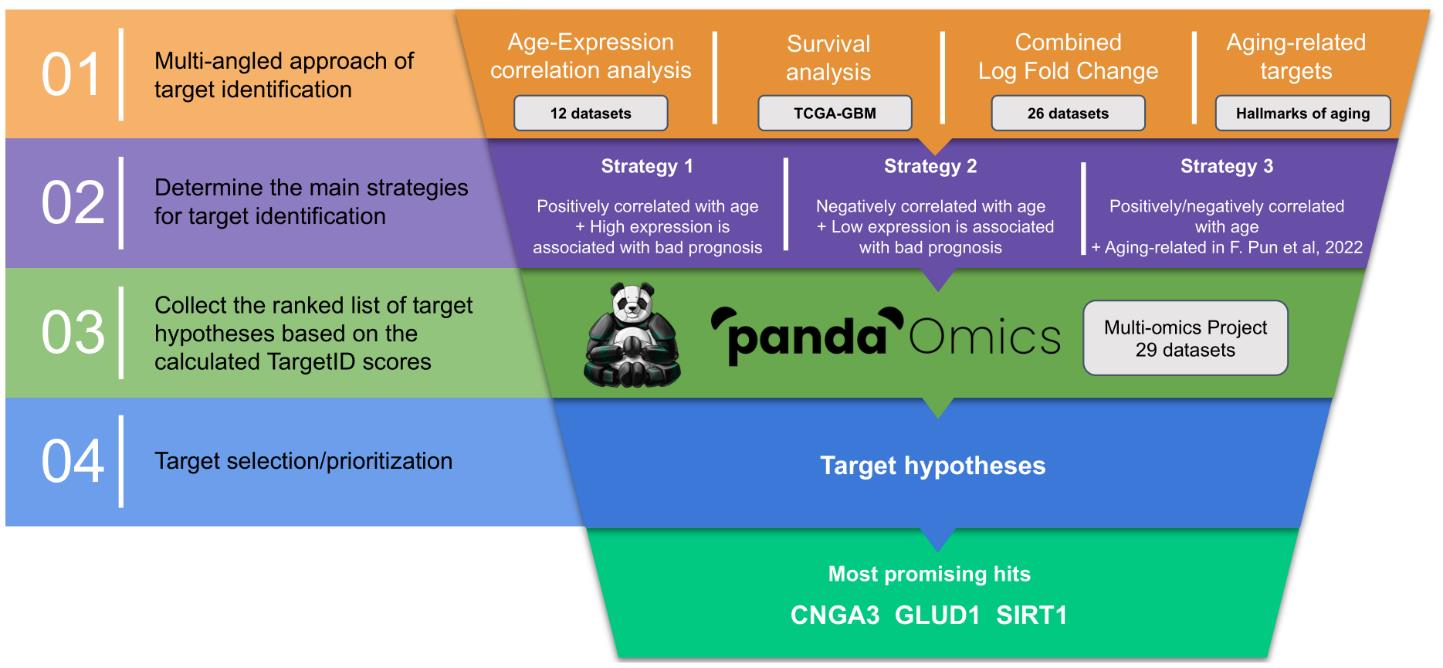
**Overall workflow of the study.** Current pipeline is designed to prioritize dual-purpose therapeutic targets by combining several data modalities following three distinct strategies of target identification. Potential target hypotheses are ranked using AI-driven scores obtained via PandaOmics TargetID engine and information regarding the combined expression, druggability, safety, novelty and accessibility by small molecules.

The starting point of our analysis was to collect relevant GBM datasets and combine them into a single multi-omics project inside the PandaOmics system. The final project consisted of data obtained from 29 different studies: 25 RNA-seq/microarray, 3 methylation and 1 proteomics (Supplementary Table 1). These data were subsequently used to generate genesets mined from age correlation analysis, survival rates, differences in expression levels and previously published information on aging-related genes [12]. Intersection of those sets following three different strategies and ranking the resulting lists by PandaOmics TargetID AI-driven scores led us to the identification of the most promising dual-purpose therapeutic target candidates (Figure 1).

### Correlation analysis

Among the list of 25 transcriptomics datasets, age metadata were available for 12 which were consequently used for the correlation analysis (Supplementary Table 2). Spearman’s correlation coefficients between gene expression and age, as well as corresponding *p*-values, were calculated for each gene.

**Figure 2:**
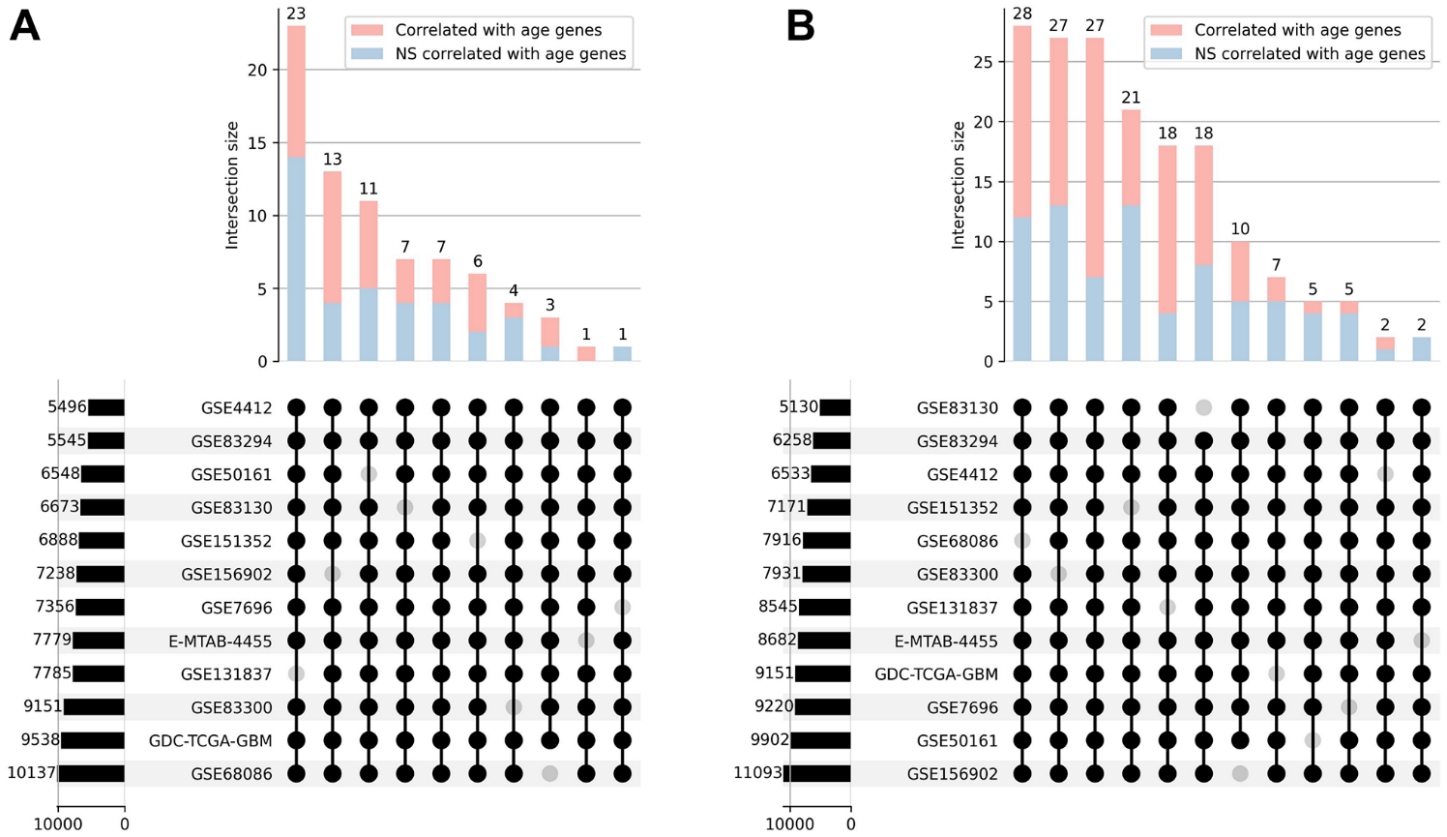
**Correlation analysis.** UpSet plots [30] representing the overlap of positively (**A**) and negatively (**B**) correlated with age genes across 12 transcriptomic datasets. The combination matrix identifies the intersections, while the bars on top represent the size of each intersection divided into significantly and not significantly (NS) correlated genes. Bars on the left depict the overall amount of correlated genes in each dataset.

Our next step was separating the genes whose expression was positively and negatively correlated with age. Intersections between gene lists from 12 datasets were performed and only those genes that correlated with age and had the same correlation sign in at least 11 out of 12 datasets were selected for further analysis. This resulted in 76 genes that were positively correlated with age and 170 that were negatively correlated with age (Figure 2). Furthermore, we applied Stouffer’s method for combining *p*-values to calculate the significance of the correlation coefficients. Only 38 out of 76 genes were significantly (Stouffer’s combined *p*-value < 0.05) positively correlated with age, and 92 out of 170 genes appeared to be significantly negatively correlated with age.

### Survival analysis

**Figure 3:**
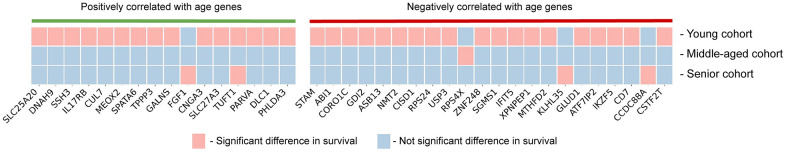
**Survival analysis results.** Each of the genes that is significantly correlated with age (n = 38) was tested for significant or not significant difference in survival rates with respect to the high and low expression levels in young, middle-aged and senior patient cohorts from TCGA-GBM dataset.

We performed a survival analysis for the genes that were significantly correlated with age, using the TCGA-GBM [29] dataset. First, we divided patients into three cohorts: young (< 45 years), middle-aged (from 45 to 60 years) and senior (> 60 years). Then for each cohort, we performed survival analysis according to the expression level of the analyzed gene. 16 out of 38 significantly positively correlated genes and 22 out of 92 significantly negatively correlated genes were able to stratify patients by survival according to their expression level (Figure 3). It is important to mention that most genes were able to significantly stratify the cohort of young patients.

### Target selection strategies guided by AI-powered PandaOmics TargetID engine

Finally, we determined three main strategies for target identification and utilized the PandaOmics TargetID engine in order to prioritize the best target hypotheses based on predictive AI-scores and additional assessment in terms of druggability, safety and brain tissue-specific expression.

### Strategy 1: Expression and survival analysis of positively-correlated with age genes

**Figure 4:**
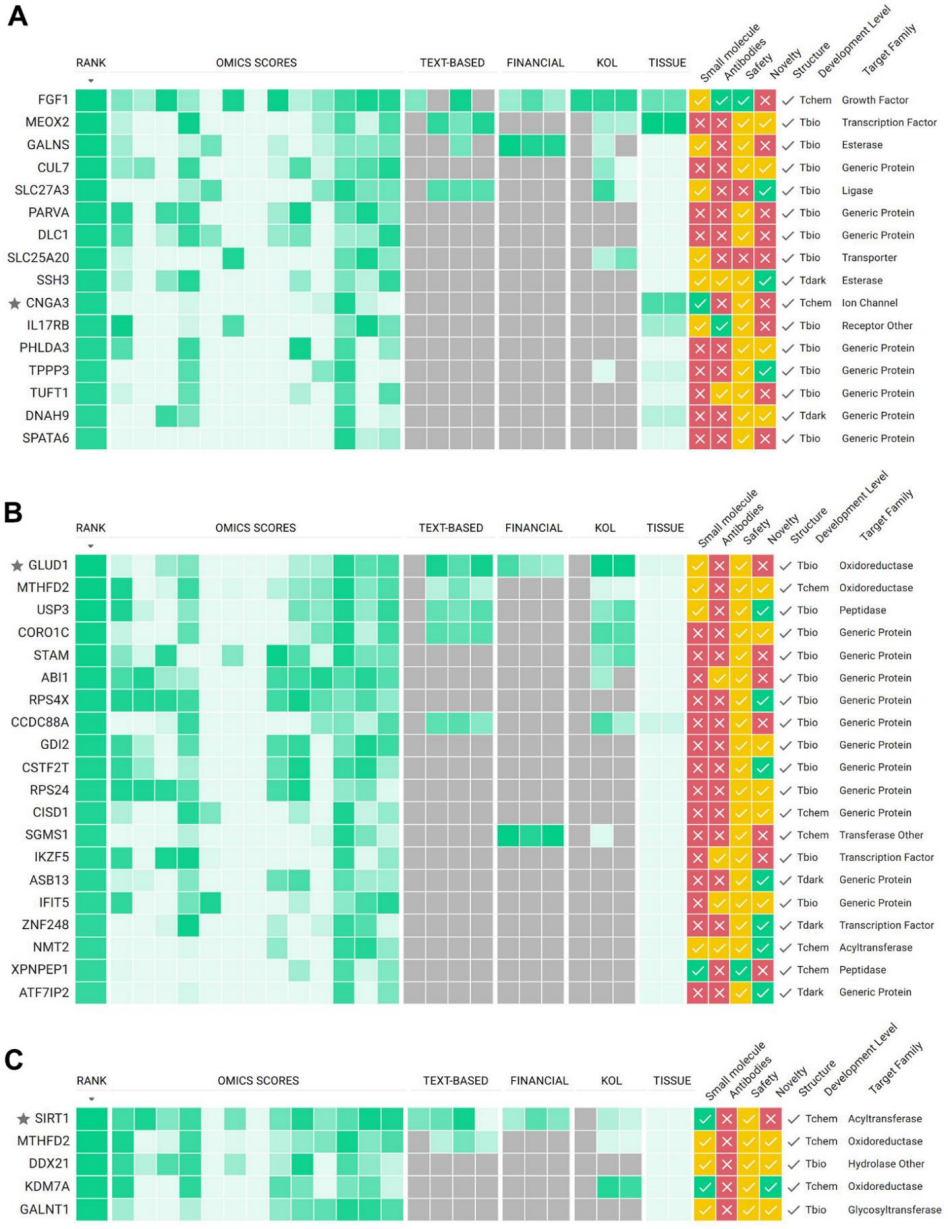
PandaOmics TargetID scoring approach for potential targets selected according to strategy 1 (**A**), strategy 2 (**B**) and strategy 3 (**C**). Target hypotheses are ranked according to the scores obtained from different AI-powered predictive models: omics-, text-, key opinion leaders (KOLs) and funding- based. For each target additional information on tissue specific expression, accessibility by small molecules and antibodies, safety, novelty, structure availability, development level and protein family are provided. For a detailed description of all scores and filters see Materials and Methods section, as well as the user manual at https://insilico.com/pandaomics/help.

The first strategy involved finding targets that met the criteria of being positively correlated with age and which high expression is significantly associated with a worse survival rate. This approach assumed that inhibiting such targets could bring benefits to GBM patients. We were able to find a set of 16 genes that satisfy both requirements and passed those to the PandaOmics TargetID engine. After manual curation of the results, we nominated *CNGA3* as a putative target. Although *CNGA3* did not top the list of the most promising targets based on PandaOmics predictions (Figure 4A), it was highly ranked by the Expression score (score value 0.91). In fact, *CNGA3* gene was significantly upregulated in 16 out of 26 expression datasets and had a positive combined log fold change of 1.47 (FDR corrected *p*-value < 0.01, Supplementary Figure 1A). Additional arguments in favor of *CNGA3* choice were its high accessibility by small molecules, specific expression in brain tissue and absence of the safety red flags.

### Strategy 2: Expression and survival analysis of negatively-correlated with age genes

The second strategy represented an inverted approach: we sought genes that were negatively correlated with age and whose low expression was associated with bad prognosis, in an attempt to find a target whose activation could benefit GBM patients. A set of 20 genes matching these conditions was analyzed and *GLUD1* was nominated as a putative target since it had the highest rank in PandaOmics TargetID analysis (Figure 4B). Additionally, we observed significantly decreased *GLUD1* levels in 15 GBM datasets which were reflected in a negative combined log fold change of -0.68 (FDR corrected *p*-value < 0.01, Supplementary Figure 1B).

### Strategy 3: Intersecting age-correlated GBM genes with potential aging targets

The third strategy was to investigate whether the age-correlated genes from GBM patients identified in this study are related to aging in general. For this, we intersected our set of 130 age-correlated genes with the list of previously identified dual-purpose disease and age-associated targets associated with the Hallmarks of aging predicted by Pun et al. [12]. The authors performed a comprehensive analysis using a variety of target identification and prioritization techniques offered by the PandaOmics platform and proposed a list of promising aging-associated targets that may be used for drug discovery. Five genes were identified in the intersection and all of them were identified as negatively correlated with age in GBM patients, namely *MTHFD2, DDX21, KDM7A, GALNT1* and *SIRT1*. The latter was nominated as a putative target following PandaOmics TargetID ranking (Figure 4C).

## DISCUSSION

Recent advances in the application of AI-powered algorithms in the target discovery field has proven to be a robust and viable method, which was demonstrated in several recent studies focused on cancer and aging research [12, 31]. However, the search for dual-purpose therapeutic targets implicated in both conditions simultaneously is still a challenge. Below we give the assessment of the targets revealed by a multi-angled AI-guided approach of target identification based on the previously published data.

### CNGA3

Among genes that are significantly positively correlated with age in GBM patients and whose high expression is associated with worse survival, we selected *CNGA3*.

*CNGA3* codes for an ion channel, which belongs to the cyclic nucleotide-gated cation channel family. *CNGA3* is involved in visual signal transduction and is essential for the generation of light-evoked electrical responses in photosensitive cones, located in the retina of the eye.

Our results are consistent with the research of Pollak et al. who showed that high expression of *CNGA3* was significantly associated with reduced median survival in GBM patients. By contrast, when the authors examined gene expression patterns in the Ivy Glioblastoma Atlas Project (Ivy GAP) database, and considered expression restricted to the central solid tumor region, high *CNGA3* was associated with increased, rather than decreased, survival. The authors suggest that this discrepancy may reflect the complexity of ion channel functions in GBM, as well as differences in sample composition between databases and bias introduced by restricting the Ivy GAP analysis to the central tumor region [32].

In another study [33], *CNGA3* was mentioned among the genes whose overexpression was associated with worse GBM patients survival and provides a predictive value of nitrosoureas treatment resistance.

In summary, *CNGA3* may play a dual role through its effects on GBM development and progression as well as aging. Further investigation is needed to confirm our hypothesis and explore specific molecular mechanisms of *CNGA3* involvement in both processes.

### GLUD1

In the group of genes significantly negatively correlated with age in GBM patients and low expression is associated with bad prognosis, the *GLUD1* gene had the highest rank in our PandaOmics TargetID analysis.

*GLUD1* encodes mitochondrial glutamate dehydrogenase 1 - a mitochondrial matrix enzyme which catalyzes the conversion of L-glutamate into alpha-ketoglutarate. In the nervous tissue, *GLUD1* is involved in healthy learning and memory creation by increasing the turnover of glutamate, an excitatory neurotransmitter. Distorted *GLUD1* function plays a role in several psychiatric and neurological disorders [34].

Several studies focusing on glutamate metabolism changes during aging in the brain demonstrated that there is a gradual rise in extracellular glutamate in the brain and an increase in the sensitivity of certain neurons to the cytotoxic effects of glutamate during aging [35, 36]. The decline in neuronal function during aging may result from increases in extracellular glutamate, glutamate-induced neurotoxicity, and altered mitochondrial metabolism [37].

In another study, Franco et al. investigated the protein expression profile of the key regulators of glutaminolysis, including *GLUD1*, in a cohort of astrocytomas of different malignancy grades and non-neoplastic brain samples. *GLUD1* expression was shown to be downregulated in GBM, and upregulated in lower grades of astrocytoma (AGII-AGIII). Significant low *GLUD1* protein levels were observed in the mesenchymal subtype of GBM. The authors suggested that the downregulation of *GLUD1* in GBM increased the source of glutamate for glutathione synthesis and enhanced tumor cell fitness due to increased antioxidative capacity [38].

### SIRT1

*SIRT1* is among the most studied genes in aging research. *SIRT1* was identified as a target connected to all hallmarks of aging, due to its wide range of interactions with aging-associated pathways [12].

*SIRT1* encodes the NAD-dependent protein deacetylase sirtuin-1 that links transcriptional regulation directly to intracellular energetics and participates in the coordination of several separated cellular functions such as the cell cycle, response to DNA damage, metabolism, apoptosis and autophagy.

The significance of the sirtuin pathways in longevity has been extensively reported [39]. *SIRT1* activity was found to be attenuated in the cerebellum during aging, leading to alterations of the epigenetic landscape, thereby changing gene expression that interferes with motor function [40]. In addition, activation of *SIRT1* suppressed aging by ensuring telomere integrity [41], antagonizing oxidative stress [42], regulating nutrient signaling by inhibiting the mTOR pathway [43] and mitochondrial unfolded protein response [44].

*SIRT1* is extensively studied in the context of cancer as well. Accumulating evidence has recently revealed that *SIRT1* may act as a tumor suppressor in several types of cancer, thus, activating *SIRT1* would represent a possible therapeutic strategy. It was recently identified as a key prognostic factor in brain cancer. A small-molecule activator of *SIRT1* showed a therapeutic potential on GBM *in vitro* and *in vivo* by inducing autophagy and mitophagy [45].

## CONCLUSIONS

Our study provides an example of a pipeline development intended to identify dual-purpose therapeutic targets. This is achieved by using the synergy of several data modalities and *in silico*-based approaches. In this regard, the application of AI-powered algorithms, such as PandaOmics, may accelerate subsequent gene target discovery not only for GBM but for a broader range of age-associated diseases. Through the three selected strategies as well as combining the GBM correlation analysis with survival analysis and AI-proposed GBM targets, we identified three potential therapeutic targets: *CNGA3, GLUD1*, and *SIRT1*, which we propose to investigate in further studies. The next steps towards implementation of the identified therapeutic targets into the clinic would involve a generation of small molecules and their optimisation with further validation and preclinical testing to determine their safety, efficacy, and potential side effects.

## MATERIALS AND METHODS

### Data collection

Gene expression data originally from Gene Expression Omnibus, ArrayExpress, and PRIDE were collected in PandaOmics, an AI-driven target discovery platform. Twenty-five transcriptomics (GSE86202, GSE151352, GSE59612, GSE68086, GSE156902, GDC-TCGA-GBM, GSE103227, GSE90886, GSE22866, GSE50161, GSE4290, GSE108474, GSE10878, GSE90598, E-MTAB-3892, GSE83130, GSE68848, GSE153746, GSE42656, GSE7696, GSE119102, GSE65626, GSE72269, GSE13276, GSE15824), 3 methylations (GSE60274, TCGA-GBM, GSE123678) and 1 proteomics (PXD017943) datasets with a total number of 2,627 samples (case = 2,027, control = 600) were analyzed. All omics datasets were pre-processed according to the PandaOmics pipeline, which automatically defines data type and normalizes the data for further analysis. Upper-quartile normalization and log2-transformation were applied for all transcriptomics datasets used. All named datasets were further used for target prioritization and hit identification using PandaOmics TargetID approach (see “PandaOmics TargetID platform for target prioritization” section). Collected transcriptomics and proteomics datasets were used for differential expression analysis (see differential expression analysis and combined log-fold changes section). Finally, 12 out of 25 transcriptomics datasets (GSE156902, GSE68086, GSE83130, GSE50161, GSE151352, GDC-TCGA-GBM, GSE7696, GSE4412, GSE83294, E-MTAB-4455, GSE131837, GSE83300) were used to conduct a correlation analysis (see age-correlation analysis section) as age metadata were available only for these datasets.

### Differential expression analysis and combined log-fold changes

Differential expression analysis was performed in PandaOmics using the *limma* R package. Each dataset has been processed according to *limma* standard protocols. Obtained gene-wise *p*-values were corrected by the Benjamini-Hochberg procedure. Combined log-fold changes (LFC) between transcriptomics and proteomics datasets were calculated in the meta-analysis section of PandaOmics. The meta-analysis section in PandaOmics enabled us to calculate combined LFC values and Q-values across all datasets used for the analysis, using minmax normalization for LFC values and Stouffer’s method combining *p*-values with further FDR correction.

### Age-expression correlation analysis

Normalized gene expression matrixes along with patients’ age metadata were collected from PandaOmics for 12 transcriptomics datasets. Spearman’s rank correlation coefficients between gene expression and age, as well as corresponding *p*-values, were calculated for each gene separately for each dataset using only case samples. Lists with positively and negatively correlated genes were analyzed and plotted independently using the *upsetplot* python package with min_degree = 11. In order to calculate the significance of the correlation coefficients across all datasets, Stouffer’s method was applied for previously obtained Spearman’s correlation *p*-values. Finally, 2 lists of genes significantly correlated with age (Stouffer’s combined *p*-value < 0.05) were obtained.

### Survival analysis

Survival analysis was performed for the genes that were significantly correlated with age, obtained from PandaOmics GDC-TCGA-GBM dataset. Patients were divided into three cohorts: young (< 45 years), middle-aged (from 45 to 60 years) and senior (> 60 years) and for each cohort survival analysis was performed. Briefly, survival analysis was performed using the KaplanMeierFitter function from *lifelines* python package. The Median function was applied to normalize gene expression data and the median value for each gene of interest was used as a threshold for patients’ stratification. Patients with the expression value of the gene of interest ≥ or < than the median value were considered as patients with “high” or “low” expression of a particular gene, respectively. Log-rank test was used to calculate the statistical significance. The probability of survival outcome for each cohort was plotted on a heatmap using *seaborn* python package and colored as red if there was a significant difference in survival between patients with high and low expression of a gene, and colored blue if there was no significant difference.

### PandaOmics TargetID platform for target prioritization

The *in silico*-based PandaOmics TargetID approach was performed on all collected omics datasets, including transcriptomics, methylomics and proteomics, to prioritize and identify the most promising GBM therapeutic targets from the lists of genes obtained through different strategies. This approach is based on combining multiple ranking scores derived from text and omics data. Text-based scores represent how strongly a particular target is associated with a disease based on sources like scientific publications, grants, patents, clinical trials and key opinion leaders. Omics scores are based on differential expression, GWAS studies, somatic and germline mutations, interactome topology, signaling pathway perturbation analysis algorithms, knockout/overexpression experiments and more omics-data sources and, thus, represent the target-disease association based on molecular connections between the proposed target and disease of interest. Regardless of the methodology, all models output ranked lists of target hypotheses. The combination of described scores leads to a ranked list of potential targets for a disease that can be filtered out based on their novelty, safety, accessibility by molecule or antibodies and availability of PDB structure. Detailed descriptions of all scores and filters can be found in PandaOmics’ User Manual (https://insilico.com/pandaomics/help). For the current study, lists with potential targets derived from strategies 1, 2 and 3 were combined and passed into TargetID. To identify the most promising hits, additional filtering was performed on PandaOmics TargetID page. After filtering, the list with the most promising hits was obtained.

## Supplementary Material

Supplementary Figure 1

Supplementary Tables
