# Supplementary material for: Identification of dual-purpose therapeutic targets implicated in aging and glioblastoma multiforme using PandaOmics - an AI-enabled biological target discovery platform
Source: Aging (Albany NY). 2023 Apr 26;15(8):2863–76. doi: 10.18632/aging.204678 (PMC10188351; doi:10.18632/aging.204678)
Supplement: Supplementary Figure 1 [file aging-15-204678-s001.pdf]

SUPPLEMENTARY FIGURE

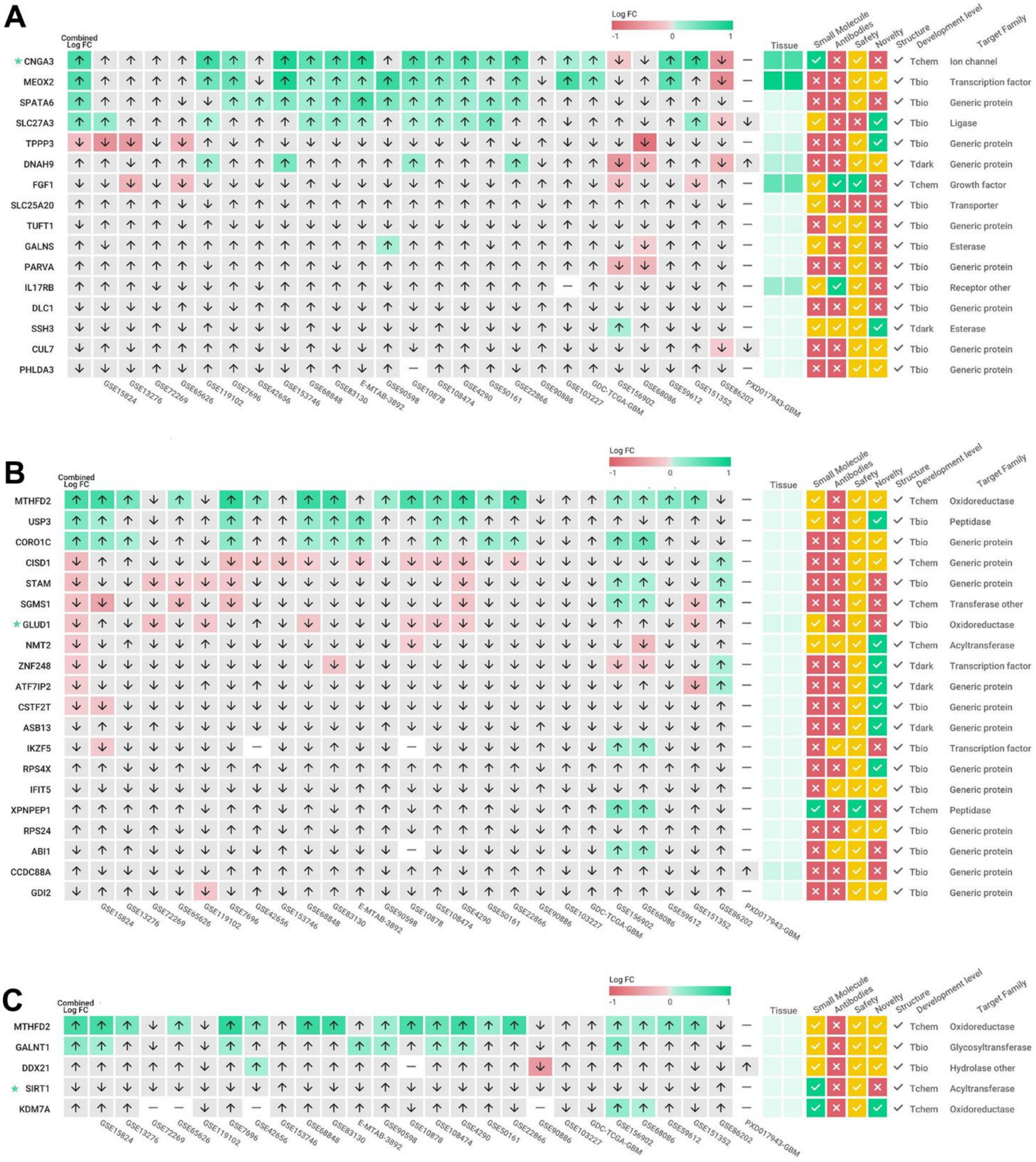

Supplementary Figure 1. Combined expression analysis for the genes obtained according to strategy 1 (**A**), strategy 2 (**B**) and strategy 3 (**C**). Combined logarithmic fold-changes (Log FC) and Q-values across all gene expression datasets are obtained using minmax normalization for Log FC values and Stouffer's method combining p-values with further FDR correction. Significant combined Log FC values represent both magnitude and direction of gene expression changes between disease and control across multiple datasets.
