# Supplementary material for: Identification of dual-purpose therapeutic targets implicated in aging and glioblastoma multiforme using PandaOmics - an AI-enabled biological target discovery platform
Source: Aging (Albany NY). 2023 Apr 26;15(8):2863–76. doi: 10.18632/aging.204678 (PMC10188351; doi:10.18632/aging.204678)
Supplement: Supplementary Tables [file aging-15-204678-s002.pdf]

SUPPLEMENTARY TABLES

Supplementary Table 1. Glioblastoma multiforme datasets used for PandaOmics analysis.

Dataset ID	Type	Year	Organism	Tissue	Total samples	Cases	Controls
TCGA-GBM	methylation	2015	Homo sapiens	brain	155	153	2
GSE60274	methylation	2014	Homo sapiens	brain	77	68	5
GSE123678	methylation	2018	Homo sapiens	brain	78	59	8
GSE83130	microarray	2016	Homo sapiens	brain	701	530	20
GSE7696	microarray	2007	Homo sapiens	brain	84	80	4
GSE42656	microarray	2013	Homo sapiens	brain	73	5	16
E-MTAB-3892	microarray	2016	Homo sapiens	brain	179	11	9
GSE15824	microarray	2011	Homo sapiens	brain	45	15	2
GSE90598	microarray	2017	Homo sapiens	brain	25	16	3
GSE10878	microarray	2008	Homo sapiens	brain	23	19	4
GSE108474	microarray	2018	Homo sapiens	brain	550	228	28
GSE68848	microarray	2015	Homo sapiens	brain	580	228	28
GSE4290	microarray	2006	Homo sapiens	brain	180	77	23
GSE50161	microarray	2013	Homo sapiens	brain	130	34	13
GSE22866	microarray	2011	Homo sapiens	brain	46	40	6
GSE90886	microarray	2016	Homo sapiens	brain	18	9	9
GSE13276	microarray	2009	Homo sapiens	brain	15	5	7
GSE65626	microarray	2015	Homo sapiens	brain	12	3	3
GSE103227	microarray	2017	Homo sapiens	brain	10	5	5
GSE72269	microarray	2015	Homo sapiens	brain	9	4	2
TCGA-GBM	RNA-seq	2015	Homo sapiens	brain	173	166	5
GSE59612	RNA-seq	2014	Homo sapiens	brain	92	39	17
GSE151352	RNA-seq	2020	Homo sapiens	brain	24	12	12
GSE153746	RNA-seq	2020	Homo sapiens	brain	21	6	3
GSE86202	RNA-seq	2016	Homo sapiens	brain	6	3	3
GSE119102	RNA-seq	2018	Homo sapiens	brain	6	4	2
GSE156902	RNA-seq	2020	Homo sapiens	blood	600	156	252
GSE68086	RNA-seq	2015	Homo sapiens	blood	285	40	55
PXD017943	Proteomics	2020	Homo sapiens	brain	66	12	54

Supplementary Table 2. Glioblastoma multiforme datasets used for correlation analysis.

Dataset ID	Type	Year	Organism	Tissue	Total samples	Cases	Controls	Cases with age	Controls with age	Age range cases
GSE83130	microarray	2016	Homo sapiens	brain	701	530	20	196	0	7-89
GSE7696	microarray	2007	Homo sapiens	brain	84	80	4	80	0	26-70
GSE4412	microarray	2006	Homo sapiens	brain	170	59	0	59	0	18-82
GSE83294	microarray	2016	Homo sapiens	brain	170	59	0	59	0	18-82
E-MTAB-4455	microarray	2017	Homo sapiens	brain	52	52	0	52	0	18-72
GSE131837	microarray	2019	Homo sapiens	brain	52	52	0	52	0	11-75
GSE83300	microarray	2016	Homo sapiens	brain	50	50	0	50	0	18-68
GSE50161	microarray	2013	Homo sapiens	brain	130	34	13	23	13	3-73
TCGA-GBM	RNA-seq	2015	Homo sapiens	brain	173	166	5	165	0	21-89
GSE151352	RNA-seq	2020	Homo sapiens	brain	24	12	12	12	12	17-73
GSE156902	RNA-seq	2020	Homo sapiens	blood	600	156	252	151	248	21-79
GSE68086	RNA-seq	2015	Homo sapiens	blood	285	40	55	39	46	11-84
